# Our approach for out-of-center initiation of extracorporeal membrane oxygenation and subsequent interhospital transport

**DOI:** 10.1007/s00508-024-02469-4

**Published:** 2024-11-22

**Authors:** Alexander Hermann, Peter Schellongowski, Oliver Robak, Nina Buchtele, Bernhard Nagler, Martin Müller, Thomas Staudinger

**Affiliations:** 1 Intensive Care Unit 13i2, Department of Medicine I, Medical University of Vienna, Währinger Gürtel 18–20, 1090 Vienna, Austria; 2 Johanniter-Unfall-Hilfe in Österreich, Ignaz-Köck-Straße 22, 1210 Vienna, Austria

**Keywords:** Extracorporeal life support (ECLS), ECMO retrieval, Mobile ECMO, Interhospital transfer, Intensive care transport

## Abstract

Extracorporeal membrane oxygenation (ECMO) initiation at a non-ECMO-capable facility by specialized mobile teams aims for a stabilization prior to center admission, internationally referred to as ECMO retrieval. It is a recommended strategy to avoid primary interhospital transfer of compromised patients with a high risk of life-threatening incidents and potentially death. Deploying the unique skill set of ECMO installation and transportation to an unfamiliar environment, however, adds a further degree of complexity to the demanding fields of both transporting the critically ill and ECMO management itself. Although recommendations for the initiation of ECMO retrieval programs exist, centers globally tailor their course of action to local individual needs and so do we.

The purpose of this work is to portray the decision-tree-based protocol of the intensive care unit 13i2 (Department of Medicine I, Medical University of Vienna) with its operational standards for optimal patient selection and transport organization.

## Background

Extracorporeal membrane oxygenation (ECMO) is a widely used treatment option for life-threatening respiratory or circulatory failure. In states of most severe gas exchange disorders, such as acute respiratory distress syndrome (ARDS), venovenous (VV) ECMO has been shown to decrease mortality [1, 2]. Venoarterial (VA) ECMO serves as a rescue option during cardiopulmonary resuscitation (CPR), also known as extracorporeal CPR (eCPR) and is further used to support the circulation in states of refractory cardiogenic shock [3–5].

Both the relatively invasive procedure of ECMO placement and the ongoing operation tend to be the objective of experienced high-volume centers with expertise in implementation, management and troubleshooting of the full extracorporeal life support spectrum [6–8]; however, transfer of patients with severely impaired gas exchange or circulation to these centers may be hazardous and bears the risk of unforeseeable complications and potentially death [6, 9–12]. The International ECMO Network as well as the Extracorporeal Life Support Organization (ELSO) therefore advise to create mobile ECMO teams within the framework of tertiary care centers, emphasizing their specific role in times of pandemic challenges [13, 14]. These expert teams visit the patient bedside and implement ECMO at the referring hospital when needed, followed by attendant interhospital transfer to the tertiary care center. This procedure is internationally referred to as ECMO retrieval.

Centers around the world tailor these recommendations for ECMO retrieval to meet their individual needs leading to globally varying modes of action [15]. The intensive care unit (ICU) 13i2 of the Department of Medicine I, Medical University of Vienna, is a listed ELSO member offering long-standing expertise in extracorporeal gas exchange treatment and providing facilities for retrieval since 2019. A decision-tree-based protocol includes operational standards for decision making, transport organization and personnel as well as material resources management.

The purpose of the present work is to give insights into our standards and thus provide accessible knowledge for primary, secondary as well as other tertiary institutions. By doing so, we aim to enhance both understanding and collaboration within the involved experts, ultimately improving the overall coordination of mobile ECMO processes in Austria.

## Aim of the program

The aim of this program is to improve patient care by bringing material and expertise to locations where they are not readily available. The present concept serves as a standardized procedural protocol for all professionals involved comprising guidance for referring institutions, defined transport logistics, a pathway for eligibility assessment for both ECMO and ECMO retrieval, quality management and continuous education.

## Organizational structure

The ICU 13i2 is an 8‑bed tertiary care medical ICU subordinated to the Department of Medicine I, Medical University of Vienna and located at the General Hospital Vienna. Physicians working in our ICU hold board certification for internal medicine with subspecialization in intensive care medicine/critical care. The ECMO retrieval teams usually consist of one intensive care senior physician/attending (ECMO team leader), one intensive care resident/fellow (ECMO assistant physician) and one ECMO-experienced certified nursing professional with completed intensive care training (ECMO nurse). The different types of team composition are delineated in detail within the ECMO retrieval process section.

Ground-based team and equipment transport is conducted with a team transport vehicle of the organ transport service under the auspices of St. John’s Ambulance Austria (Johanniter-Unfall-Hilfe in Österreich, hereinafter Johanniter). Air-bound team and equipment transport as well as secondary interhospital patient transfer is individually organized in collaboration with one of the suitable institutions.

Regular theoretical and practical training sessions as well as simulations are mandated for all staff members involved in the program and explicitly include the team of the emergency team transport vehicle. Within these training sessions, we address both ECMO management and transport-associated details. Substantial information referring to changes in our course of action are distributed real-time by means of a mailing list and encrypted instant messaging services.

## ECMO retrieval process

**Fig. 1:**
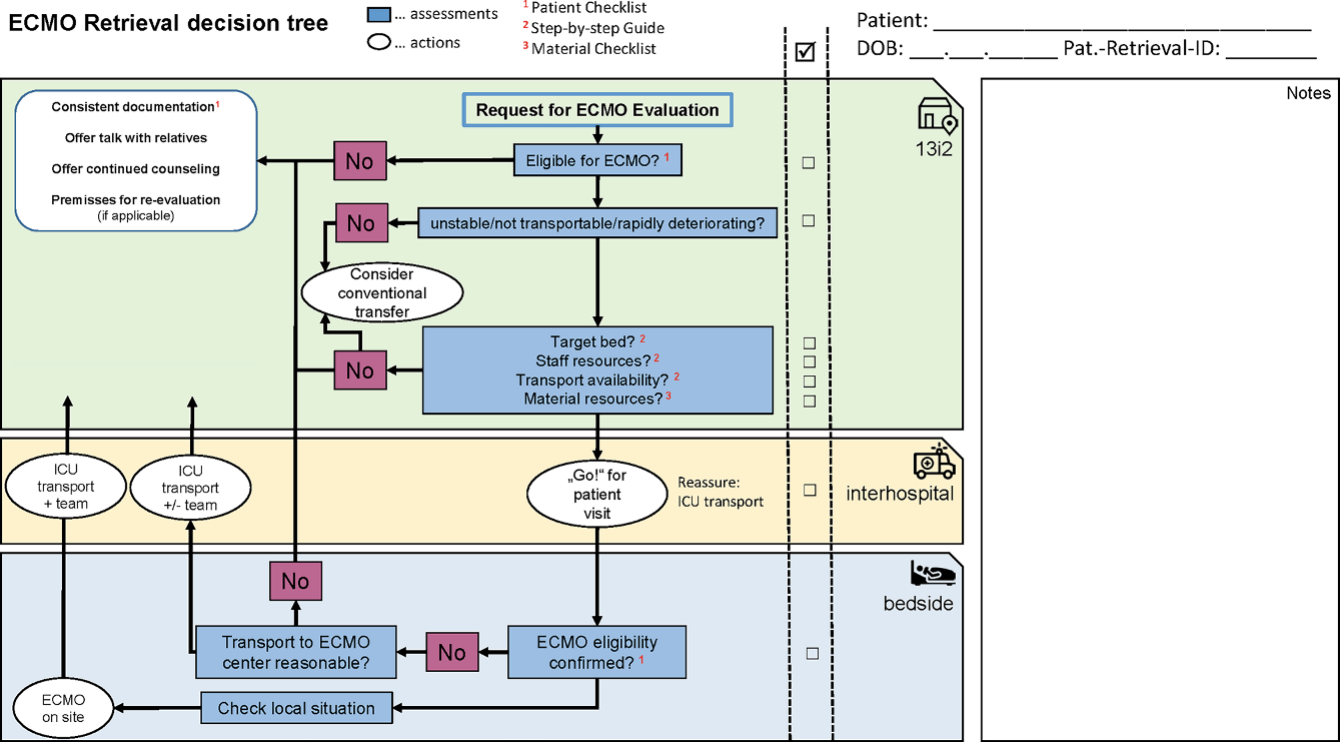
ECMO retrieval decision tree

The process of ECMO retrieval is illustrated in Fig. 1. Predominantly, we offer primary transports for adults, which means that the mobile team performs ECMO cannulation at the referring institution and then accompanies the patient to our center. When needed, we also conduct secondary and tertiary transport for adults as defined by ELSO [16].

### Request and evaluation

**Fig. 2:**
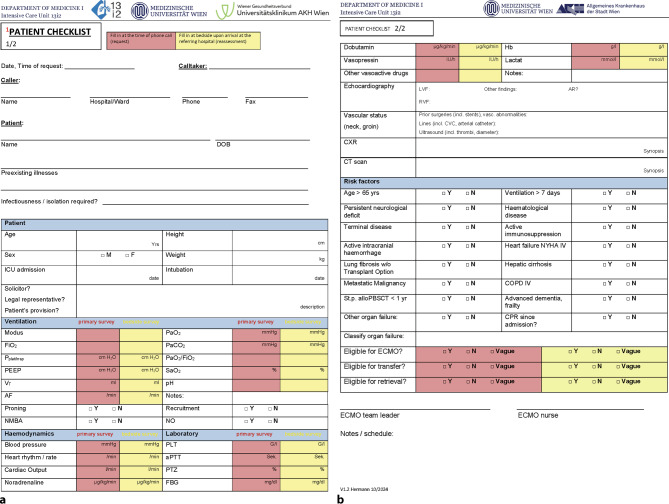
**a** Patient checklist page 1. **b** Patient checklist page 2 *DOB* date of birth, *ICU* intensive care unit, *FiO2* fraction of inspired oxygen, *Pplat/insp* Plateau/inspiratory pressure, *PEEP* positive end-exspiratory pressure, *VT* tidal volume, *AF* respiratory rate, *NMBA* neuromuscular blocking agents, *PaO2* arterial oxygen partial pressure, *PaCO2* arterial carbondioxide partial pressure, *SaO2* arterial oxygen saturation, *NO* nitric oxide, *PLT* platelets, *aPTT* activated partial thromboplastin time, *PTZ* prothrombin time, *FBG* fibrinogen, *Hb* hemoglobin, *LVF* left ventricular function, *RVF* right ventricular function, *AR* aortic regurgitation, *incl.* including, *vasc.* vascular, *CVC* central venous catheter, *CXR* chest X-ray, *CT* computed tomography, *yrs* years, *w/o* without, *alloPBSCT* allogeneic peripheral blood stem cell transplantation, *yr* year, *NYHA* New York Heart Association, *COPD* chronic obstructive pulmonary disease, *CPR* cardiopulmonary resuscitation, *ECMO* extracorporeal membrane oxygenation, *Y* yes, *N* no, *V* version.

Referring institutions may be ICUs, emergency departments and intermediate care units; however, emergency transport companies may also request evaluation for ECMO retrieval. Contact details as well as basic information about the program are published online (https://www.muv.ac.at/13i2). Requests are usually received by telephone. Each case is discussed individually in a conversation between the attending senior physician at ICU 13i2 and the treating physician at the referring institution. We regard the use of our adapted patient checklist based on our own evaluation sheet [17] as mandatory (see Fig. 2a, b) to meet our demands for both evaluation and documentation standards.

Candidacy for ECMO treatment and ECMO retrieval are assessed separately.

### Indications for ECMO

We primarily consider patients for respiratory ECMO including the subcategory of extracorporeal CO_2_ removal. The evaluation of respiratory ECMO indication itself adheres to our standard in-house principles complying with the ELSO Patient Care Practice Guidelines [18–20] and the European Society of Intensive Care Medicine (ESICM) guidelines [21] summarily shown in Table 1. We also assess patients for cardiac ECMO following the respective VA ECMO guidelines [4, 18, 19, 22]; however, except for unforeseen emergency situations, retrieval options for VA ECMO patients remain a focus for future development. For the final rule-in we always take definite decisions on an individual patient-centered basis. When the ECMO indication is borderline, the attending senior physician involves peer experts to discuss the case. In patients eligible for bridging strategies such as to organ transplantation, we involve the cardiac or thoracic surgeon on call into the decision-making process.

**Table 1:** Indications for VV ECMO in adult patients according to ELSO guidelines [20] and ESICM guidelines [21]. Both presume potential reversibility of respiratory failure and optimal conservative management

ELSO guidelines	ESICM guidelines
Hypoxemic respiratory failure (Horovitz index of < 80 mm Hg) *or* Hypercapnic respiratory failure (pH < 7.25 with inspiratory plateau pressure > 35 cm H_2_O or prolonged refractory hypercapnia with pH < 7.25 with a PaCO_2_ ≥ 60 mm Hg). *or* Respiratory support as a bridging solution to lung transplantation or primary graft dysfunction following lung transplantation	Hypoxemic respiratory failure (Horovitz index of < 50 mm Hg for > 3 h) *or* Hypoxemic respiratory failure (Horovitz index of < 80 mm Hg for > 6 h) *or* Hypercapnic respiratory failure (pH of < 7.25 with a PaCO_2_ of ≥ 60 mm Hg for > 6 h using an increased respiratory rate up to 35 breaths per minute and a plateau pressure of ≤ 32 cm H_2_O)

*ELSO* Extracorporeal Life Support Organization, *ESICM* European Society of Intensive Care Medicine, *Horovitz* PaO_2_/FiO_2_ ratio, *VV ECMO* venovenous extracorporeal membrane oxygenation

### Indications for ECMO retrieval

The paramount goal remains to assess the patient as early as possible and, if feasible, to offer a safe center admission without antecedent intervention by a mobile ECMO team. The ECMO retrieval is generally considered when the risk of a conventional transport is deemed unacceptable. The candidacy for retrieval is impacted by clinical factors, transport-related circumstances and the critical appraisal of all clinicians involved. We regard states of circulatory and/or respiratory instability, no transport option without ECMO, and rapid deterioration as rule-in criteria. Among others, this may be the case in severe gas exchange disorders, such as a Horovitz index of < 50 mm Hg despite optimal conservative management, pre-cardiac arrest situations or antecedent cardiopulmonary resuscitation (CPR) due to the underlying condition, need to reposition the patient from prone to supine position for transport although thereby provoking imminent instability, transport denial by an emergency medical physician, high vasopressor doses or hyperlactatemia. Also, we reflect the degree of pretransport hypoxemia, hypoperfusion, and acidosis on the expected duration of transport to anticipate the risk of further deterioration arising from the transport duration itself; however, the consensus of the entire teams at the referring institution, the mobile ECMO team and the ICU team remaining in-house are a sine qua non for the conduction of an ECMO retrieval.

When potentially reversible pathologies such as untreated pneumothorax are contributing to the patient’s condition, the retrieval is delayed, and the candidacy is re-evaluated after the cause has been addressed.

Currently, we do not routinely launch to patients in cardiac arrest to perform eCPR; however, when time from conventional CPR start to the expected time of our team’s arrival is below 30 min, and eCPR criteria are met, we continue our retrieval process with the aim of emergency VA cannulation. This may particularly be the case in patients already accepted for retrieval, who develop cardiac arrest during our approach. In these cases, we adhere to our in-house emergency department’s recommendations [22].

Principally, we also extend our portfolio to scenarios which may necessitate the change of an already running ECMO circuit or a modification in cannula configuration prior to the transport.

### Resource management

Upon acceptance for ECMO retrieval and reassurance of the available target bed, we assess our own unit’s staff resources for ECMO retrieval which includes considerations for both the outreach team and the ICU team remaining in-house. The latter must be able to meet the clinical demands even when unexpected events occur during the absence of the retrieval team. To provide retrieval availability even during peak times or personnel shortages, we expanded our capabilities for ECMO retrievals with a pool of ICU nursing staff. This initiative allows us to mostly guarantee the participation of at least one nurse capable of managing both intensive care nursing and ECMO management.

When ECMO retrieval cannot be offered due to any reason, the hazards of a primary transport to our center without antecedent ECMO implementation will be taken after careful consideration.

### Preparation

After a positive decision to retrieve the patient, the preparation phase ensures that 1) the mobile ECMO team is assembled and informed, 2) equipment is packed and checked, 3) transport for both team approach and ICU transfer is organized and 4) the referring institution is briefed.

### Team assembly

The assembly of the retrieval team is done by the ECMO team leader in collaboration with either the 13i2 head of nursing, if present, or with the most experienced ECMO nurse in charge.

**Fig. 3:**
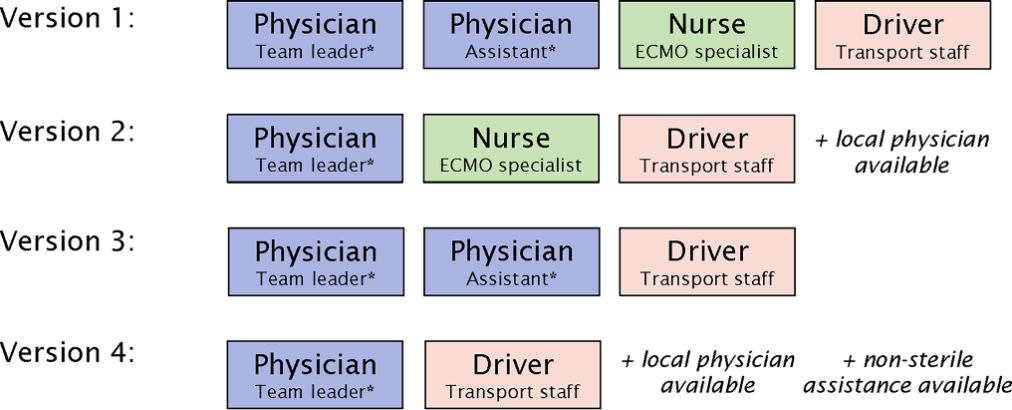
Mobile ECMO team versions dependent on staffing. Asterisk Can be the cannulation provider

The team composition is flexible depending on personnel resources and transport factors. It allows four different versions when a ground-based approach is conducted (see Fig. 3). For an air-bound transport, team compilation is adapted to the air ambulance operator’s individual specifications. The option with one physician only necessitates the availability of medical capacities at the referring institution (at least one physician or nurse for sterile guided assistance during cannulation), which is ascertained during the telephone conversation. The same requirement applies when no ECMO nurse is available for transport.

Self-protection by means of personal protective equipment according to our institution’s hygiene standards and reflective vests for the interhospital period is provided.

### Packing procedure

Our transport equipment is mainly prepacked and located in the ICU. The remaining items are individually added. The ECMO-specific sterile goods are enclosed in duplicate. We include a versatile handheld ultrasound device as a back-up. For retrieval purposes, Cardiohelp® (Maquet, Getinge Group, Rastatt, Germany) consoles are used comprising sensors for real-time pressure and blood measurements. Alternatively, we use Xenios (Xenios AG, Heilbronn, Germany) devices. We prefer the transport of ready primed ECMO circuits to the patient to keep preparation time at the referring institution as short as possible. Depending on the travelling distance, patient-related factors and weather conditions, an ECMO heat exchange device is added.

### Transport

When a ground-based approach is performed, equipment and ECMO team are transported to the referring hospital by a designated emergency team transport vehicle of the Johanniter. It is exclusively available for medical and equipment transfers on a 24/7 basis. Each vehicle provides seats for five team members plus driver and a 220 V power supply. The Johanniter emergency coordination center uses a specified transport code for ECMO retrieval purposes ensuring correct dispatch of one of the dedicated vehicles regardless of the person answering the request. The air-bound approach is individually organized to meet all aeronautical and medical demands.

Before the mobile ECMO team launches, intensive care transport availability for the subsequent patient transfer is checked. For this purpose, we use a prioritized list including 10 organizations offering intensive care transfer capabilities (6 of which are land-based and 4 are air-bound). This list also includes the respective information about individual response time, place of departure, oxygen and air supply, 220 V power supply, solution for ECMO fixation and other specifics, such as seats available in the case of full attendance. Before the mobile team launches, the selected organization is prealerted and confirmation of transfer capacity is obtained. Ultimate alerting is done by the referring hospital subsequently to ECMO implantation.

### Briefing of the referral institution

**Fig. 4:**
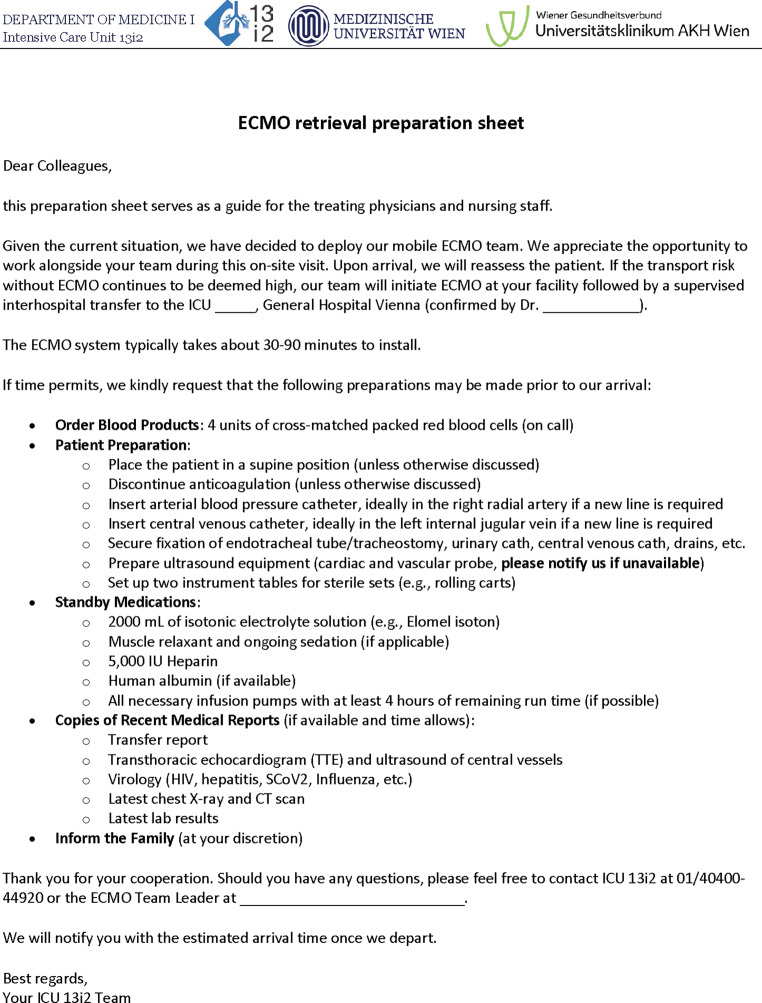
Preparation sheet for the referral hospital

During the telephone conversation with the referring institution, information about the upcoming ECMO retrieval is provided. Subsequently, a 1-page preparation sheet is sent to the institution (Fig. 4). It serves as an information source containing key points about the upcoming retrieval and including specific preparation recommendations, such as ordering packed red blood cells at call or preparation of the ultrasound machine.

### Conduction of the ECMO retrieval

After completion of the preparation process delineated above, a final team and equipment check is done together using the “Let’s-go” checklist shown in Table 2. Then, the mobile ECMO team launches.

**Table 2:** “Let’s-go” checklist

Immediately prior to departure towards referral hospital
ECMO circuit preprimed for < 4 weeks?
ECMO circuit deaerated?
4 plastic clamps and 2 metal clamps attached? Blood pump set to 0 rpm?
Priming bag filled with > 1.5 l isotonic solution?
Priming bag deaerated?
Batteries full? Power cable present?
O_2_ supply: normal function and filled with > 200 bar?
Reserve O_2_ supply (if applicable): normal function and filled with > 200 bar?
Telescope bar present? (unrig prior to vehicle loading)
3‑way lines connected to ECMO circuit?
Mobile telephones of team members present? (numbers exchanged?)
Drinking water/snacks?

*ECMO* extracorporeal membrane oxygenation,* rpm* revolutions per minute

### Approach

As soon as the emergency team transport vehicle is heading for the referring hospital, information about the expected time of arrival is provided to the referring institution by telephone. This conversation is also used to exchange updates about the patient’s condition. All members of the mobile ECMO team are updated about the current situation and the patient’s condition including the most probable strategies for ECMO configuration, cannula implantation and alternative solutions for an unexpected deterioration as well as potential back-up capacities (e.g., local cardiac catheterization laboratory for additional guidewires).

### Arrival

Upon arrival and introduction, the team splits: the ECMO team leader reviews the diagnostic findings together with the attending doctor of the referring institution to confirm the ECMO indications. Simultaneously, the ECMO assistant physician performs a preliminary echocardiography assessment to estimate cardiac function, volume status and valve function. This protocol also includes exploratory sonography of both pleural spaces and abdomen. Then, bilateral vascular sonography is performed of both groin and neck veins and arteries including diameter measurements. Also, simultaneously the ECMO circuit as well as the console are checked by the ECMO nurse and preparation of the cannulation is started. When the indications for ECMO and ECMO retrieval remain unchanged, all parties involved are informed and briefed. These steps may be shortened or partly omitted when the patient’s condition does not allow any time delay.

### Cannulation

According to our idea of an optimal retrieval, the procedure for ECMO implantation itself should deviate as little as possible from the familiar protocols followed in-house; however, when placing ECMO in an unfamiliar environment with possible back-up limitations for complications, we aim for a maximum margin of safety (also see “Risk mitigation” section).

Once arrangements are completed, role allocation and chronological order of the steps are fixed and communicated. Cannulation is done using Seldinger’s technique under real-time sterile ultrasound guidance. We do not routinely provide surgical techniques such as cut down for cannulation under vision. Typically, we serially place the guidewires after ultrasound-guided puncture, followed by sonographic reassurance of the correct wire position. When the patient’s coagulation is within normal limits, heparin is given as a bolus of 50 IU/kg bodyweight at the earliest when both cannulas are in place. Further reassurance of the correct cannula positions may be obtained by means of blood gas analysis at any time point or after circuit connection by opening all clamps prior to blood flow application to detect unexpected backflow due to cannula displacement; however, as mentioned previously, the detailed methods of operation are subject to the personal experience of the cannulation providers and should follow their habitual style as close as possible.

We routinely start ECMO with an initial blood flow of 1 lpm and a sweep gas flow of 1 Lpm for the first minute, unless the patient’s condition requires an instant increase of blood flow. We determine optimal settings primarily using monitoring of the vital parameters, arterial and venous blood gas analysis (BGA), end-tidal CO_2_ (etCO_2_), echocardiography as well as clinical judgement.

The preparation for departure begins with ECMO start. The first section of the “back-to-base” checklist ensures that all necessary actions are taken immediately after ECMO start (Table 3).

**Table 3:** “Back-to-base” checklist

**Immediately after ECMO start**
Blood pressure and ECMO pressures suspecting cannula misplacement?
Color difference of pre-oxygenator and post-oxygenator tubing? (*If no: check patient, gas line, O*_*2*_* supply)*
Ventilator adapted? *(e.g. protective ventilation)*
Systemic anticoagulation administered? *(e.g. heparin bolus)*
Blood products for transport needed? *(e.g. Hb <* *7* *g/dl with short, <* *8* *g/dl with long transport time, ongoing bleeding)*
Body core temperature normal? (*If no, counteract with blankets, heaters etc.)*
ECMO sensor calibration with pre-oxygenator ABG done?
Essential interventions necessary? *(e.g. limb perfusion cannula, arterial line at A. rad. dext.)*
Interhospital intensive care transport alerted?
**Immediately prior to departure towards destination ICU (patient already on the stretcher)**
VV ECMO blood flow approx. 60–80% of estimated CO?
ECMO sweep gas flow adapted to last ABG?
Emergency gear crank/reserve pump motor accessible?
2 ECMO clamps and 1 ECMO scissors to everyone’s accessibility?
ECMO battery status ok?
All tubes and lines (blood and gas) exposed and w/o compression/kinks?
Capnography and capnometry established? etCO_2_ matched with PaCO_2_?
Alarm limits including real time Hb and temperature set?
Information to ICU 13i2 about estimated time of arrival, potential diagnostic stop-over discussed (e.g. CT scan)?
Material complete? *(emergency bag* *+* *cannula kit* *+* *documentation* *+* *back-up ECMO set)*

*VV ECMO* venovenous extracorporeal membrane oxygenation,* Hb* hemoglobin,* ABG* arterial blood gas analysis,* ICU* intensive care unit, *CO* cardiac output,* etCO2* end-tidal carbon dioxide,* PaCO*_*2*_ arterial partial pressure of carbon dioxide, *CT* computed tomography, *A. rad. dext.* right radial artery

### Departure and interhospital transfer

The interhospital transfer vehicle is alerted according to the previously arranged workflow.

During another telephone call with the destination ICU, the expected time of arrival is announced and both the necessity and possibility of a diagnostic stopover such as for a contrast-enhanced computed tomography (CT) are discussed and arranged when appropriate.

Once the patient is on the stretcher and prepared for departure, the second section of our “back-to-base” checklist (Table 3) ensures that our transport prerequisites are met. Usually, the ECMO team leader and the ECMO nurse accompany the patient inside the interhospital transfer vehicle together with at least one paramedic. The emergency team transport vehicle transports the remaining team members and the equipment not in use. It follows in convoy or travels individually when air-bound transfer is conducted.

### Completion

After arrival and handover procedure, the retrieval is finalized with the completion of the documentation and a team debriefing. Equipment is restocked and prepared for the next transport. Short-term and long-term feedback possibilities are offered for all parties involved.

## Risk mitigation

We acknowledge the risks arising from ECMO placement at external facilities. Particularly in the absence of immediate specialized surgical support at peripheral hospitals, complications could pose a significant risk to patient outcomes. We addressed this by implementing a combination of the following strategies for risk mitigation.

### Prior assessment of cannulation risk

During the evaluation process, we aim for early identification of patients with higher risks of vascular complications such as prior surgeries, vascular abnormalities, or difficult anatomical access (Fig. 2b). Additionally, thorough ultrasound assessment before, during, and after cannulation evaluates vascular integrity and identifies potential challenges for cannula placement at all possible access sites. When high risk of complications prior to cannulation advises against out-of-center ECMO initiation, the hazards for a conventional transport can be taken upon individual decision.

### Checklist-based protocol

Among other features, our protocol emphasizes safety by integrating checkpoints through the use of mandatory checklists, such as immediately after cannulation (Table 3), enabling the early identification of complications.

### Risk management and training

Disinfection and aseptic exposure are routinely carried out for all four entry sites (i.e., neck and groin bilaterally) to avoid any time delay when cannula placement turns out to be futile at one site. Patient blood management includes the advance ordering of cross-matched packed red blood cells on call (Fig. 4). When the hemoglobin concentration is ≤ 7 g/dl after ECMO initiation with an expected transport duration of less than 30 min, or ≤ 8 g/dl for transport durations of 30 min or more, or if active bleeding is present, packed red blood cells may be requested from the referring institution for transfusion during transport. As a prophylactic measure, we administer systemic anticoagulation at the earliest when the correct cannula position is confirmed via ultrasound. When bleeding complications with an imminent threat arise during the retrieval process, individualized management enables either expedited transfer to the center or the involvement of surgical assistance at the referring hospital. As previously mentioned, our advanced simulation training is provided for all team members including scenarios for handling complications during cannulation.

### Perspectives

Despite all our endeavours to minimize risks, however, peri-interventional complications can never be entirely excluded. Therefore, our decisions regarding ECMO and ECMO retrieval will continue to be primarily guided by an individualized risk-benefit assessment.

Future developments of our program may include a stronger focus on cardiac ECMO retrieval, along with on-call agreements with the collaborating departments.
